# Significance of Combining Bronchoalveolar Lavage Fluid With Targeted Next‐Generation Sequencing in the Pathogen Detection‐Based Diagnosis of Pulmonary Infections

**DOI:** 10.1111/crj.70046

**Published:** 2025-01-21

**Authors:** Jiangbo Liu, Bo Yang, Yu Wu, Guihong Yang, Xiaojiu Zha, Wei Jiang

**Affiliations:** ^1^ Department of Pulmonary and Critical Care Medicine Tianjin First Central Hospital Tianjin China; ^2^ Department of Thoracic Surgery Tianjin First Central Hospital Tianjin China; ^3^ Department of Pulmonary Diseases Dafang County Traditional Chinese Medicine Hospital Bijie Guizhou China; ^4^ Infectious Diseases Department Tianjin First Central Hospital Tianjin China

**Keywords:** bronchoalveolar lavage fluid, conventional microbiological tests, metagenomic next‐generation sequencing, pulmonary infection, targeted next‐generation sequencing

## Abstract

**Objective:**

In this study, we investigated the application value of bronchoalveolar lavage fluid (BALF) combined with targeted next‐generation sequencing (tNGS) in the pathogen detection‐based diagnosis of patients with lung infections.

**Method:**

A retrospective analysis was conducted on patients who underwent tracheoscopy and conventional microbiological tests (CMTs) on BALF, coupled with metagenomic next‐generation sequencing (mNGS) or tNGS. This investigation encompassed individuals with suspected lung infections at Tianjin First Central Hospital from March 2023 to July 2023. Diagnostic rates based on pathogens detected via tNGS were compared with CMTs within the tNGS group. Additionally, diagnostic rates obtained through tNGS were compared with mNGS between the two groups.

**Results:**

The data of a total of 169 patients (78 in the tNGS group and 91 in the mNGS group) were collected, and 145 patients (67 in the tNGS group and 78 in the mNGS group) were finally diagnosed with lung infections. The comprehensive positive pathogen detection‐based diagnosis rate for tNGS was 86.6%, with a single‐pathogen lung infection diagnosis rate of 85.7% and a mixed‐pathogen pulmonary infection diagnosis rate of 88.0%. In contrast, the overall positive pathogen detection‐based diagnosis rate for CMTs was 38.8%, comprising a single‐pathogen pulmonary infection diagnosis rate of 28.6% and a mixed‐pathogen pulmonary infection diagnosis rate of 20.0%. The difference in positive diagnosis rate was deemed statistically significant (*p* < 0.05). In the mNGS group, the overall pathogen detection‐based diagnosis rate was 89.7%, with a single‐pathogen pulmonary infection diagnosis rate of 84.9%, and a 100% diagnosis rate for mixed‐pathogen pulmonary infections. There was no statistically significant difference in the positive diagnosis rate when compared with the tNGS group (*p >* 0.05).

**Conclusion:**

In patients with pulmonary infections, the diagnosis rate based on BALF pathogen detection using tNGS exceeded that of CMTs, showing no statistically significant difference compared to mNGS.

## Graphical abstract

This study demonstrates the superior diagnostic efficacy of bronchoalveolar lavage fluid (BALF) combined with targeted next‐generation sequencing (tNGS) in identifying pathogens associated with lung infections compared to conventional microbiological tests (CMTs). The results suggest that tNGS is a valuable tool for accurate and comprehensive diagnosis, comparable to metagenomic next‐generation sequencing (mNGS) in this context.

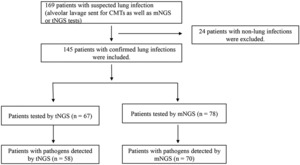

## 1 Introduction

Pulmonary infection is prevalent worldwide, with high morbidity and mortality, particularly in the elderly and immunocompromised populations [1, 2]. Furthermore, the high cost of pulmonary infection diagnosis and treatment places a significant financial burden on the patients [3]. Swift diagnosis based on rapid pathogen detection is crucial for patients with pulmonary infections, as it enables timely control and contributes to better outcomes. Early identification of the pathogenic bacteria can significantly enhance prognosis [4]. Pathogen detection methods typically used for conventional pathogen identification include microbial culture, nucleic acid testing, molecular immunology testing, and pathologic diagnosis. However, due to the recognized limitations of traditional pathogen detection methods, such as the narrow range of pathogen detection and the lack of timeliness and sensitivity, the above methods cannot satisfy clinical needs in terms of pathogen diagnosis [5]. In recent years, a rapid and wide‐coverage pathogen diagnosis method for the detection of pathogenic microorganisms has been developed, namely, metagenomic next‐generation sequencing (mNGS), which has transformed the pathogen detection‐based diagnosis pattern for critical and seldom‐treated infections. It is advantageous in the diagnosis and management of pneumonia in immunocompromised patients [6], severe pneumonia [7], pediatric pneumonia [8] and can quickly identify the pathogen and guide the treatment. However, mNGS has certain shortcomings, such as its high cost, as it needs to be performed by a third‐party testing agency; therefore, it cannot be covered by health insurance and cannot simultaneously screen DNA and RNA viruses [9]. In recent years, with the rapid development of molecular diagnostic technology, we can currently adopt a faster, more economical, and more accurate technology‐targeted sequencing (tNGS) to achieve early diagnosis of respiratory infections. Through targeted PCR amplification and high‐throughput sequencing, pathogens can be quickly identified within 24 h, greatly reducing diagnostic time. At the same time, the cost is lower than mNGS [9]. Targeted next‐generation sequencing (tNGS) is a rapid and cost‐effective test for pulmonary infection. In this study, we aim to assess the diagnostic efficacy of tNGS for pathogen identification and compare it with conventional microbiological tests (CMTs) and mNGS in terms of pathogen detection‐based diagnosis rates.

## 2 Study Participants and Methods

### 2.1 Study Participants

**FIGURE 1:**
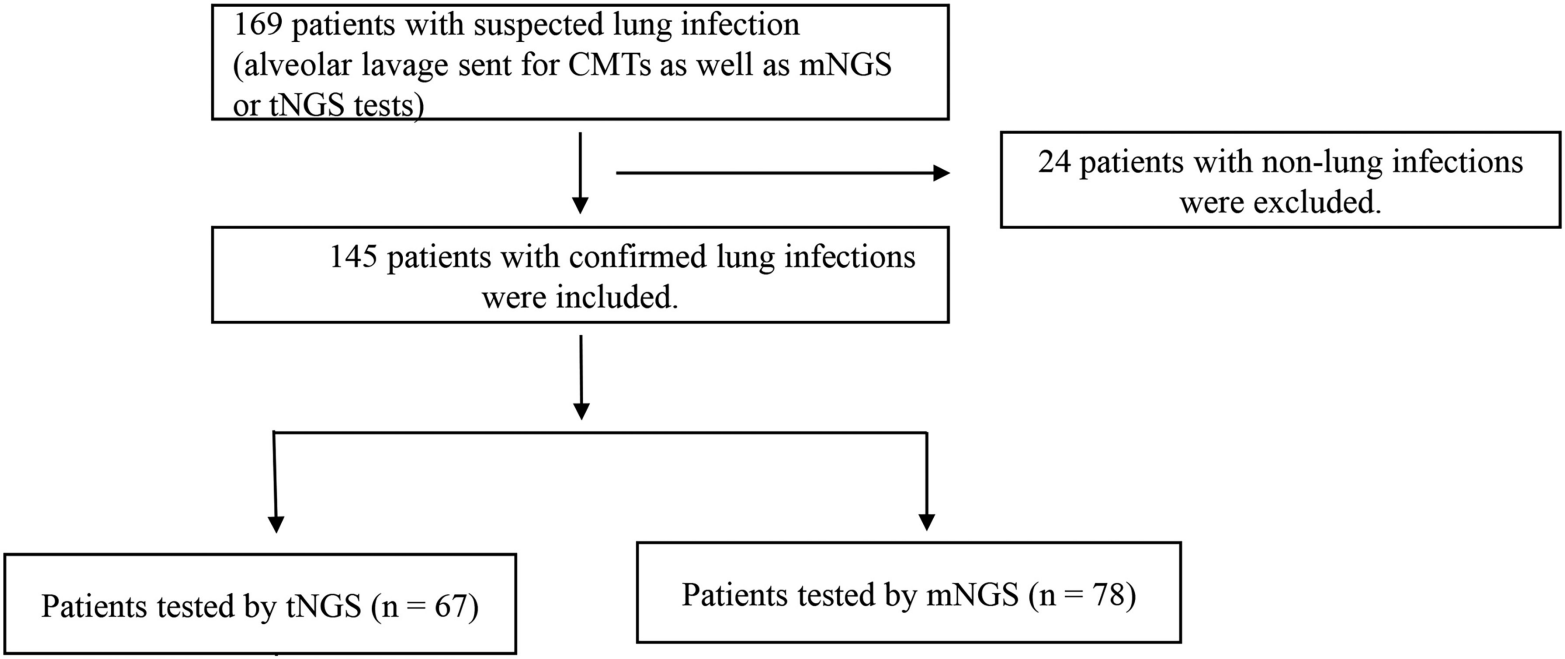
Study flow chart.

A retrospective analysis was conducted on 169 patients who underwent tracheoscopy and bronchoalveolar lavage fluid (BALF) examinations for suspected pulmonary infections at Tianjin First Central Hospital from March 2023 to July 2023 (Figure 1). The BALF from all patients underwent CMTs, including culture, smearing, real‐time polymerase chain reaction (RT‐PCR), antigen–antibody testing, and histopathology examination, as well as mNGS or tNGS. After excluding 24 patients with noninfectious conditions, the study cohort comprised 145 patients, with 67 in the tNGS group and 78 in the mNGS group. This study was approved by the institutional review committee.

### 2.2 Inclusion and Exclusion Criteria

#### 2.2.1 Inclusion Criteria

(1) presence of respiratory symptoms such as fever, cough, sputum production, and difficulty breathing; (2) chest imaging examination shows newly appearing patchy infiltrating shadows, consolidation shadows of lobes or segments, ground glass shadows, or interstitial changes; (3) patients with indications for bronchoscopy examination and retention of lavage fluid for relevant laboratory tests; and (4) patients can be followed up regularly for at least 1 month.

#### 2.2.2 Exclusion Criteria

(1) patients aged < 18 and > 80 years old; (2) patients with incomplete clinical data; (3) unable to tolerate bronchoscopy examination; (4) patients clinically diagnosed as nonpulmonary infections; and (5) patients who could not be contacted during the follow‐up process.

### 2.3 Study Methods

#### 2.3.1 Data Collection

We recorded basic data, chest computed tomography (CT) features, lesion location, stage of the bronchoscope entering the trachea, microbiological test results, tNGS and mNGS results, and pathologic results for each included patient. The final clinical diagnosis was determined by posttreatment response and a 1‐month follow‐up.

#### 2.3.2 Tracheoscopy

Bronchoscopy was conducted in the Respiratory Endoscopy Center by the same experienced endoscopist. All patients conformed to the indications for bronchoscopy, and BALF, lung biopsy tissues, and other specimens were collected from lung lesions; the lesions were located using the thin‐layer CT, virtual navigation, hand‐drawn navigation, and bronchial ultrasonography. In the case of a single lesion, the lung specimens were collected from the lesion; in the case of multiple lesions, the lung specimens were collected from larger lesions with connected trachea, and in the case of a diffuse lesion, the specimens were collected from the typical lesions. Lobular bronchi were defined as the primary bronchi. A portion of BALF was allocated for pathogen identification through culture, smearing, polymerase chain reaction (PCR) test, and Xpert analysis. A remaining 5 mL was reserved for mNGS or tNGS.

#### 2.3.3 tNGS and mNGS Processes

##### 2.3.3.1 tNGS Process

Sample preprocessing: 0.1 moL/L DTT liquefaction reagent was used for even mixing, and the solution was placed for 3 to 5 min until the sample became liquefied (sputum or viscous BALF, and this step was not required for clarified BALF). Next, 1.3 mL of the sample to be tested was taken and centrifuged at 12 000 rpm for 5 min. Subsequently, a fraction of the supernatant was extracted, and 500 μL was transferred to the grinding tube. The lysate was introduced and subjected to high‐speed blending at 4900 rpm for 60 s, pausing for 20 s, repeated twice, with an interval; the vibration cycle was performed thrice (180 s). Following cell wall disruption, the mixture was centrifuged at 12 000 rpm for 5 min for subsequent utilization.

Nucleic acid extraction: After centrifugation, 250 μL of the sample was taken, and 20 μL of Proteinase K was added for extraction with a nucleic acid extraction or purification kit (Guangzhou KingCreate Biotechnology Co., Ltd., Art. No.: KS118‐BYTQ‐96).

Library construction: The Multiplex Combined Detection Kit for Respiratory Pathogenic Microorganisms (Guangzhou KingCreate Biotechnology Co., Ltd.) was used to concentrate 14 μL of nucleic acid. If the concentration equaled or exceeded 10 ng/μL, nuclease‐free water was added to reach 14 μL; in cases of lower measured nucleic acid concentration (below 10 ng/μL), 14 μL was directly taken. The cycle protocol comprised 1 cycle at 95°C for 3 min, 28 cycles at 95°C for 30 s, 60°C for 30 s, 72°C for 30 s, and 1 cycle at 72°C for 1 min. Subsequently, the solution was held at 4°C, and the PCR product underwent brief centrifugation before adding 25 μL of purified magnetic beads for PCR product purification in the target region. For library amplification, 11.5 μL of PCR product was collected, and an immediate centrifugation was performed. Subsequently, 25 μL of purified magnetic beads was added for library purification. The library's concentration and quality were assessed using Qubit. Sample libraries underwent pooling, and 35 μL of purified magnetic beads was added for further library purification after pooling.

Sequencing: After pooling, the library was sequenced using the Universal Sequencing Reaction Kit (Guangzhou KingCreate Biotechnology Co., Ltd., Art. No.: MR100), with a testing time of 6 h.

##### 2.3.3.2 mNGS Process

Total nucleic acid was extracted from 500 μL of BALF. A DNA sequencing library was created for each patient, and an RNA sequencing library was created specifically for the patient with a suspected viral infection. DNA was extracted following the kit instructions; the established library was purified, amplified, and repurified; and Qsep1 and Qubit were used to quantitatively test the library fragment size and library concentration, respectively. The quantitative library was then measured based on the preset amount of on‐computer data, and the Nextseq 550Dx sequencer was used for sequencing.

#### 2.3.4 Conformity Assessment

The conformity rate between clinical results and diagnosis results using tNGS, mNGS, and CMTs, as well as the conformity rate between tNGS and CMTs, was evaluated. The conformity rate between clinical results and diagnosis results refers to the conformity between tNGS, mNGS, and CMT results and chest imaging, anti‐infective therapy, and clinical outcomes. The conformity rate between tNGS and CMT test results refers to the consistency of the outcomes acquired by the two testing techniques.

### 2.4 Statistical Analysis

The SPSS 25.0 statistical software was used to process the clinical data. Normally distributed measurement data are expressed as mean ± standard deviation, and the independent sample *t* test was used for comparisons between groups. The counting data are expressed as the number of cases and a percentage, and the intergroup comparison was performed using a chi‐squared test; a two‐sided test was performed with a test level of α = 0.05.

## 3 Results

### 3.1 Comparison of Basic Clinical Data Between the Two Groups of Patients

A total of 145 patients (67 in the tNGS group and 78 in the mNGS group) were included in this study. Table 1 shows the demographic characteristics and basic clinical data of the patients in the two groups. There were no significant differences between the mNGS group and the tNGS group in baseline data such as gender, age, lesion size (in the area of tracheoscopic sampling), CT tracheal grade of the lesion, grade of tracheoscopic entry into the trachea, lesion characteristics, and the number of cases of histopathologic examination sent for testing.

**TABLE 1:** Comparison of demographic characteristics and basic clinical data characteristics between the tNGS group and the mNGS group.

	tNGS group (*n* = 67)	mNGS group (*n* = 78)	*p*
Gender (cases, male or female)	33/34	44/34	0.389
Age (year)	58.6 ± 15.6	54.3 ± 14.1	0.086
Lesion size (mm)			
≥ 30 mm	43	60	0.092
< 30 mm	24	18	
Tracheal lesion stage on CT (stage)	4.39 ± 0.95	4.51 ± 0.98	0.439
Stage of the bronchoscope entering the trachea (stage)	3.22 ± 0.90	3.35 ± 0.81	0.394
Characteristics of the lesion			
Single lesions (cases)	38	48	0.542
Multiple lesions (cases)	18	15	
Diffuse lesion (cases)	11	15	
Number of patients sent for histopathology examination (cases sent)	50/17	52/26	0.295

### 3.2 Comparison of Final Pathogen Diagnosis Results Between the Two Groups

**FIGURE 2:**
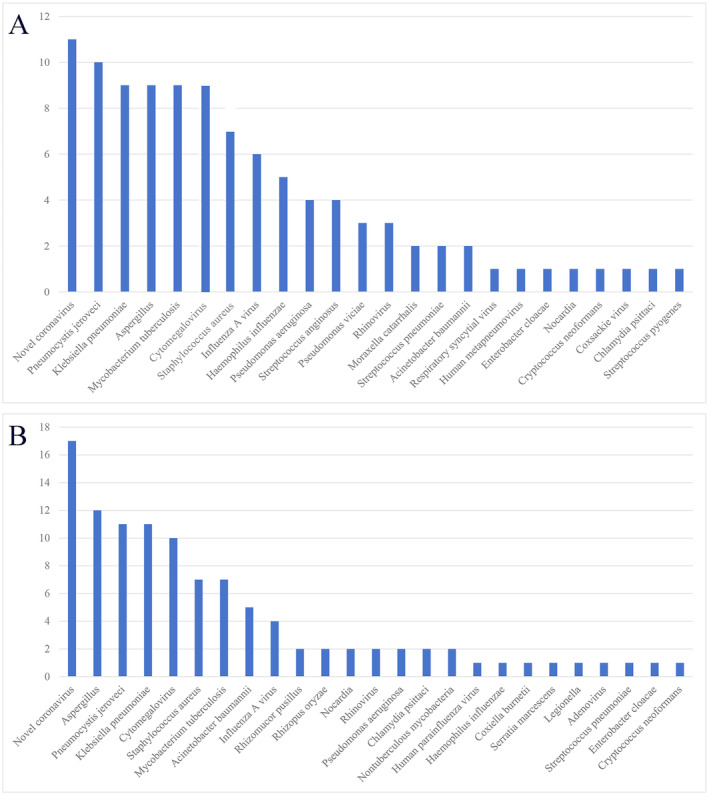
Pathogen distribution map. (A) Map of pathogen distribution detected by tNGS method. (B) Distribution map of moral pathogens detected by mNGS method.

Table 2 shows a comparison of pathogen diagnosis results between the tNGS group and the mNGS group. There was no significant difference between the two groups in the proportion of single infection and mixed infection (*p* = 0.506); in the tNGS group, 25 (37.3%) had mixed infection, and 42 (62.7%) had single infection, including 38 cases of bacterial infection, 27 cases of viral infection, 16 cases of fungal infection, 9 cases of tuberculosis infection, and 1 case of *Chlamydia psittaci* infection, whereas in the mNGS group, 25 (32.1%) had mixed infection, and 53 (67.9%) had single infection, including 32 cases of bacterial infection, 33 cases of viral infection, 27 cases of fungal infection, and 9 cases of tuberculosis infection, as well as 2 cases of *C. psittaci* infection, 1 case of rickettsia infection, and 2 cases of nontuberculous mycobacterial infection. Figure 2 depicts the distribution of pathogens in the two groups. The top five pathogens detected by tNGS were neocoronavirus 11; *Pneumocystis jirovecii* 10; *Klebsiella pneumoniae* 9; aspergillus 9; and tuberculosis 9, whereas the top five pathogens detected by mNGS were neocoronavirus 17; aspergillus 12; *P. jirovecii* 11; *K. pneumoniae* 11; and CMV10.

**TABLE 2:** Comparison of pathogen diagnosis results between the tNGS group and the mNGS group (cases [%]).

	tNGS group (*n* = 67)	mNGS group (*n* = 78)	*p*
Mixed infection (cases, %)	25 (37.3%)	25 (32.1%)	0.506
Single infection (cases, %)	42 (62.7%)	53 (67.9%)	
Diagnosis rate of different pathogens			
Bacteria 1 (cases)	38	32	
Virus 2 (cases)	27	33	
Fungus 3 (cases)	16	27	
Tuberculosis 4 (cases)	9	9	
Others 5 (cases)	*Chlamydia psittaci* 1	Chlamydia psittaci 2; coxiella burneti 1; NTM2	

### 3.3 Comparison of Pathogen Diagnosis Rates Between tNGS and CMTs in the tNGS Group

Table 3 shows a comparison of pathogen detection‐based diagnosis results between tNGS and CMTs in the tNGS group. Among the 67 patients, 58 (86.6%) were diagnosed by tNGS, including 36 cases of single infection and 22 cases (88%) of mixed infection, whereas 26 cases (38.8%) were diagnosed using CMTs, including 12 cases (28.6%) of single infection and 5 cases (20.0%) of mixed infection. As depicted in Table 3, tNGS had a higher diagnostic rate for bacterial, viral, fungal, and tuberculosis pathogens than CMTs (*p* > 0.05).

**TABLE 3:** Comparison of pathogen detection‐based diagnosis results between tNGS and CMTs in the tNGS group (cases [%]).

	tNGS (*n* = 67)	CMTs (*n* = 67)	*p*
NGS‐diagnosed cases/undiagnosed (cases [%])	58/9 (86.6%)	26/41 (38.8%)	0.000
Comparison of diagnosis rates for single infections (cases [%])	36/6 (85.7%)	12/30 (28.6%)	0.000
Diagnosed or undiagnosed mixed infection (cases [%])	22/3 (88.0%)	5/20 (20%)	0.000
Diagnostic rate of different pathogens			
Bacteria (diagnosed/undiagnosed)	31/7	10/28	0.003
Virus (diagnosed/undiagnosed)	24/3	9/18	0.000
Fungus (diagnosed/undiagnosed)	14/2	6/10	0.000
Tuberculosis (diagnosed/undiagnosed)	9/0	3/6	0.000
Other (diagnosed/undiagnosed)	chlamydia psittaci 1/0	0/1	NS

### 3.4 Comparison of Diagnosis Rates Between tNGS and mNGS

Based on the overall pathogen detection‐based diagnosis by mNGS, 70 cases (89.7%) were confirmed. There were 45 cases (84.9%) of single infection in the mNGS group and 25 cases (100%) of mixed infection, including 30 cases of bacterial infection, 32 cases of viral infection, 24 cases of fungal infection, and 7 cases of tuberculosis infection; there was no significant difference in the tNGS diagnosis rate (*p* > 0.05). In the tNGS group, pathogen‐based diagnosis failed in nine cases: one case was finally diagnosed with actinomyces and *P. jirovecii* infection by mNGS; one case was diagnosed with novel coronavirus and *P. jirovecii* infection by nucleic acid testing combined with clinical manifestations; one case was diagnosed with novel coronavirus and *Proteus mirabilis* infection by nucleic acid and culture; and one case was diagnosed with *Streptococcus pharyngitis* by BALF culture. The remaining five patients were diagnosed clinically; viral infection was considered in one case, and bacterial infection was considered in four cases. In the mNGS group, pathogen‐based diagnosis failed in eight cases, including two cases of tuberculosis (one case was diagnosed by biopsy pathology, and one case was clinically diagnosed); two cases of bacterial infection confirmed based on clinical manifestations; and one case of viral infection confirmed based on clinical manifestations; one case of cryptococcosis confirmed based on *Cryptococcus neoformans* capsular antigen and pathologic diagnosis; one case of aspergillosis confirmed based on clinical manifestations; and one case of *Hormographilla aspergillata* infection confirmed based on BALF culture.

## 4 Discussion

Traditional pathogen‐based detection methods for lower respiratory tract infections include direct microscopic observation, nucleic acid testing, culture identification, serological testing, and pathological examination; however, CMTs, such as microbial culture, are time‐consuming with low sensitivity and cannot meet clinical needs due to a lack of diagnostic tests for rare pathogens and the susceptibility to external influences [12]. Histopathology and smears can be used to identify some microorganisms, such as fungi, *M. tuberculosis* , and some bacteria, but there are severe limitations on viral diagnosis. Nucleic acid testing can be used to detect viruses, atypical pathogens (such as mycoplasma, chlamydia, and legionella), and fungi (such as *P. jirovecii*), but it can only be used to detect limited types of pathogens clinically [13]. Pathogen load, anti‐infective treatment, food and drug intervention, and specimen type can all alter the sensitivity of some CMTs.

tNGS is essentially a nucleic acid testing technology that can cover more than 95% of respiratory infections and detect common pathogens in a single specimen. In addition, it is also less expensive and more sensitive, and as a result, it can meet the need for etiological diagnosis of most clinical infectious diseases [9]. In this study, tNGS could detect respiratory viruses such as novel coronaviruses, influenza A, rhinoviruses, human metapneumoviruses, coxsackieviruses, and DNA viruses such as EBVs and cytomegaloviruses in a single specimen, but mNGS required two specimens. tNGS could also detect most fungi, including *P. jirovecii*, aspergillus, and cryptococcus; furthermore, it had a high detection rate for bacteria. The presence of respiratory viruses such as novel coronavirus and influenza A in the pathogen test could be related to the spread of COVID‐19 and influenza A epidemics, whereas the presence of aspergillus and *S. aureus* was related to secondary infections after respiratory viral infections [14, 15]. There were more immunocompromised patients in both groups, which was associated with a higher detection rate of *P. jirovecii* (Figure 2) [16].

In patients with pulmonary infections, tNGS outperformed CMTs in terms of overall diagnostic rate, single infection diagnosis rate, and mixed infection diagnosis rate (Table 3). When compared to CMTs, tNGS had obvious advantages in terms of the types and efficiency of pathogen diagnosis. Due to a limited detection range, tNGS could not detect some rare pathogens. Conducting simultaneous CMTs and tNGS would enhance the accuracy of pathogen detection, providing greater benefits to patients.

Several recent studies have demonstrated that mNGS significantly improved the pathogen detection‐based diagnosis rate of pneumonia, including severe pneumonia, immunocompromised pneumonia, and pediatric pneumonia [6, 8, 17]. Although mNGS has developed as a potential diagnostic technique for infectious diseases, greater limitations have emerged as clinical use has increased. First, it was found that the human host is the primary source of microbial nucleic acids in mNGS, constituting more than 95% of the reads. This characteristic renders mNGS less sensitive and complicates the interpretation of results [18, 19]. Second, mNGS can detect the nucleic acids of pathogens as well as colonized and contaminated pathogens, which increases the probability of false positives and easily leads to over‐treatment by clinicians. At present, modified mNGS, such as quality/quantity mNGS (QmNGS), is being used for the detection of lung pathogens; QmNGS is similar to usual mNGS (UmNGS) in terms of sensitivity. The specificity of QmNGS is higher than that of UmNGS, although the difference is not statistically significant [20]. Finally, when compared with traditional pathogen detection methods and tNGS, mNGS involves a higher financial cost, and if a patient needs to be tested for both RNA and DNA microorganisms, it increases the financial burden.

In comparison to mNGS, tNGS can detect DNA and RNA viruses, as well as distinguish subtypes of pathogens, and detect drug resistance and virulence genes while identifying the pathogens. tNGS requires less time and has a lower cost. To differentiate between contamination, colonization, and infection, tNGS data should be paired with clinical manifestations. According to the results of this single‐center retrospective study, tNGS enjoyed the same pathogen detection‐based diagnosis rate as mNGS in patients with pulmonary infections (Table 4). In recent years, mNGS has been extensively used in clinical practices in China, and most clinicians have recognized that mNGS can significantly increase the pathogen detection‐based diagnosis rate, but they have paid less attention to tNGS and studies on the pulmonary infection diagnosis rate. In this study, pathogens such as rickettsia and NTM were detected in the mNGS group but not in the tNGS group, which was thought to be due to the small sample size rather than a lack of sensitivity of tNGS as previous studies on the diagnosis of legionella and nontuberculous mycobacterial infections showed satisfactory diagnostic performance [21, 22, 23]. Moreover, clinicians have noted that pulmonary infections in numerous patients often involve a combination of multiple pathogens, posing challenges in both explanation and diagnosis of mixed infections [24]. There was no difference in single and mixed pathogen infections between the tNGS group and the mNGS group. The diagnosis rate of mixed infection by tNGS was 88.0%, and the mNGS diagnosis rate was 100% (Table 4). mNGS may offer a potential advantage for the diagnosis of mixed infections, even though the results were not statistically different. mNGS can detect a broader spectrum of pathogens and detect the pathogens more comprehensively, particularly rare pathogens. However, there was no significant difference in the overall pathogen detection‐based diagnosis rate between tNGS and mNGS. Li et al. [9] reached a similar result, claiming that tNGS was equal to mNGS in detecting pathogenic microorganisms in adult patients with pneumonia. tNGS, including the vast majority of common pathogens of lung infections. For noncritically ill patients and lung infection patients who do not consider rare pathogens clinically, tNGS can be used instead of mNGS. tNGS diagnosis of pulmonary infection pathogens is fast, accurate, and cost‐effective and is worthy of clinical promotion.

**TABLE 4:** Comparison between tNGS and mNGS diagnosis rates.

	tNGS group (*n* = 67)	mNGS group (*n* = 78)	*p*
NGS diagnosis rate (diagnosed/undiagnosed, %)	58/9(86.6%)	70/8(89.7%)	0.553
Diagnosis rate of single infection (diagnosed/undiagnosed, %)	36/6(85.7%)	45/8(84.9%)	0.912
Diagnosis rate of mixed infection (diagnosed/undiagnosed, %)	22/3(88%)	25/0(100%)	0.235
Diagnosis rate of different pathogens			
Bacteria (diagnosed/undiagnosed)	31/7	30/2	0.676
Virus (diagnosed/undiagnosed)	24/3	32/1	1.000
Fungus (diagnosed/undiagnosed)	14/2	24/3	0.509
Tuberculosis (diagnosed/undiagnosed)	9/0	7/2	0.462
Other (diagnosed/undiagnosed)	Chlamydia psittaci 1/0	Chlamydia psittaci 2/0; Rickettsia 1/0; NTM 2/0	NS
tNGS undiagnosed cases Final result (diagnostic basis)	Actinomycetes and *Pneumocystis jirovecii* 1 (mNGS retest)	Tuberculosis 2 (1 case diagnosed clinically; 1 case diagnosed by tracheoscopic biopsy)	
	Novel coronavirus and *P. jirovecii* 1 (COVID‐19 nucleic acid testing combined with clinical diagnosis)	Bacteria 2 (clinical diagnosis)	
	Novel coronavirus and *Proteus mirabilis* (COVID‐19 nucleic acid testing and lavage fluid culture)	Virus 1 (clinical diagnosis)	
	*Streptococcus anginosus* 1 (lavage fluid culture)	Cryptococcus 1 ( *Cryptococcus neoformans* capsular antigen + tracheoscopic biopsy pathology)	
	Bacteria 4 (clinical diagnosis)	Aspergillus 1 (clinical diagnosis)	
	Virus 1 (clinical diagnosis)	*Hormographilla aspergillata* 1 (lavage fluid culture)	

*Note:* Compared with metagenomic next‐generation sequencing (mNGS), this study demonstrates that bronchoalveolar lavage fluid (BALF) binding to target next‐generation sequencing (tNGS) is comparable to mNGS in identifying pathogens associated with lung infections.

There are certain limitations to this study: First, all patients underwent tracheoscopy following local anesthesia; however, some experienced false‐negative results due to poor tolerance or imprecise irrigation sites. To enhance the positivity rate, alternative methods, such as painless tracheoscopy, are recommended. Second, tNGS was conducted by a specific company, whereas mNGS results were obtained from a different company, introducing variations in pretest processing, testing methods, and positive rates. Third, due to economic constraints, specimens from the same patient were not concurrently tested by both methods for direct comparison, introducing a degree of uncertainty in the comparative results. Fourth, tNGS and mNGS had higher diagnosis rates for mixed infections than single infections, a finding that might not entirely align with real‐world scenarios. Mixed lung infections are common, but it is often difficult to accurately identify which pathogens are infections and which are colonizing or contaminating bacteria through pathogen detection results (such as culture, PCR methods, tNGS, and mNGS). In clinical practice, when identifying infecting pathogens, colonizing pathogens, and contaminating pathogens, it is often necessary to combine imaging features, clinical data, and treatment results, as well as the personal experience of doctors. It is difficult to accurately determine mixed infection pathogens, and the final results may be biased. Thus, both tNGS and mNGS have higher diagnostic rates for mixed infections than for single infections, which may not be entirely consistent with reality.

## 5 Conclusion

Detecting pathogens in the context of mixed infections poses a significant challenge, particularly in discerning whether the pathogens identified by tNGS and mNGS are indeed infectious agents. The complexity of arriving at accurate judgments is compounded by the necessity to integrate clinical features, imaging, and posttreatment outcomes for a comprehensive assessment. tNGS as a rapid, accurate, and cost‐effective alternative to mNGS for diagnosing pulmonary infections in noncritically ill patients and those where rare pathogens are not clinically considered. Lastly, given that this study was a single‐center retrospective investigation, inherent limitations, including selection bias, were unavoidable. Consequently, prospective and multicenter data are imperative for further validation and comparison.

## Author Contributions

Conception and design of the research: Jiangbo Liu and Bo Yang. Acquisition of data: Jiangbo Liu, Yu Wu, Guihong Yang, and Xiaojiu Zha. Analysis and interpretation of the data: Jiangbo Liu, Bo Yang, and Wei Jiang. Statistical analysis: Jiangbo Liu, Bo Yang, and Yu Wu. Writing of the manuscript: Jiangbo Liu, Guihong Yang, and Xiaojiu Zha. Critical revision of the manuscript for intellectual content: Jiangbo Liu, Bo Yang, and Wei Jiang. All authors read and approved the final draft.

## Ethics Statement

This study was conducted with approval from the Ethics Committee of Tianjin First Central Hospital. This study was conducted in accordance with the declaration of Helsinki. Written‐informed consent was obtained from all participants.

## Consent

The authors have nothing to report.

## Conflicts of Interest

The authors declare no conflicts of interest.

## Data Availability

The datasets used and/or analyzed during the current study are available from the corresponding author on reasonable request.
